# Mitochondrial dysfunction in neurodegenerative disorders: Potential therapeutic application of mitochondrial transfer to central nervous system-residing cells

**DOI:** 10.1186/s12967-023-04493-w

**Published:** 2023-09-09

**Authors:** Felipe A. Bustamante-Barrientos, Noymar Luque-Campos, María Jesús Araya, Eliana Lara-Barba, Javiera de Solminihac, Carolina Pradenas, Luis Molina, Yeimi Herrera-Luna, Yildy Utreras-Mendoza, Roberto Elizondo-Vega, Ana María Vega-Letter, Patricia Luz-Crawford

**Affiliations:** 1 Laboratorio de Inmunología Celular y Molecular, Facultad de Medicina, Universidad de los Andes, Santiago, Chile; 2 Centro de Investigación e Innovación Biomédica (CiiB), Universidad de los Andes, Mons. Álvaro del Portillo 12455, Las Condes, Santiago Chile; 3 Cells for Cells, Regenero, Las Condes, Santiago Chile; 4 IMPACT, Center of Interventional Medicine for Precision and Advanced Cellular Therapy, Santiago, Chile; 5 Facultad de Medicina y Ciencia, Universidad San Sebastián, Puerto Montt, Chile; 6 Laboratorio de Biología Celular, Departamento de Biología Celular, Facultad de Ciencias Biológicas, Universidad de Concepción, Concepción, Chile; 7 Escuela de Ingeniería Bioquímica, Pontificia Universidad Católica de Valparaiso, Valparaiso, Chile

## Abstract

Mitochondrial dysfunction is reiteratively involved in the pathogenesis of diverse neurodegenerative diseases. Current in vitro and in vivo approaches support that mitochondrial dysfunction is branded by several molecular and cellular defects, whose impact at different levels including the calcium and iron homeostasis, energetic balance and/or oxidative stress, makes it difficult to resolve them collectively given their multifactorial nature. Mitochondrial transfer offers an overall solution since it contains the replacement of damage mitochondria by healthy units. Therefore, this review provides an introducing view on the structure and energy-related functions of mitochondria as well as their dynamics. In turn, we summarize current knowledge on how these features are deregulated in different neurodegenerative diseases, including frontotemporal dementia, multiple sclerosis, amyotrophic lateral sclerosis, Friedreich ataxia, Alzheimer´s disease, Parkinson´s disease, and Huntington’s disease. Finally, we analyzed current advances in mitochondrial transfer between diverse cell types that actively participate in neurodegenerative processes, and how they might be projected toward developing novel therapeutic strategies.

## Introduction

Brain degeneration affect millions of persons worldwide. Globally, more than 600 people per 100,000 inhabitants over the age of 50 suffer from some form of dementia, while motor neuron diseases reach 4.5 per 100.000 populations. Mitochondrial dysfunction represents a distinctive aging hallmark, being involved in the etiopathogenesis of several disorders including brain degenerative diseases. Lately several treatments have been proposed, including the artificial replacement of dysfunctional mitochondrial by healthy isolated mitochondria (i.e. artificial mitochondrial transfer). Therefore, the present review summarizes general aspects regarding both mitochondrial structure and function, and centered the discussion on how mitochondrial dysfunction is reiteratively associated with brain degeneration. We also discuss the current status of therapies aimed to correct mitochondrial dysfunction through artificial mitochondrial donation from a healthy cell donor. Finally, we recapitulate the potential mechanism associated to the intercellular communication between the exogenous and endogenous mitochondria in the acceptor cells and provide novel perspectives on cell-free isolated mitochondria as a developing therapeutic approach for neurodegenerative disorders.

## Mitochondria

### Mitochondrial structure and function

Mitochondria is a double membrane organelle with a similar protein and phospholipid composition to the eukaryote plasma membrane. The outer membrane (OMM) communicates mitochondria with cytosolic components through various membrane complexes, including distinct forms of the translocase of the outer membrane (TOM) and mitochondrial import complex (MIM) [1, 2]. On the other hand, the inner membrane (IMM) has a longer surface area than the OMM, whose simple invagination originate the so-called *cristae* [2]. Typical crista morphology outcomes from a fine balance between intra-mitochondrial membrane fusion and fission, thus conferring structural and functional plasticity depending on the energetic status [2]. Crista organization is critical for increasing the reaction surface since respiratory chain complexes are inserted in the IMM. Furthermore, cristae junctions are stabilized through distinct proteins which are highly regulated by the Mitochondrial Contact Site and Cristae Organizing System (MICOS). It determines the cristae shape and thus the assembly and efficiency of respiratory complexes; therefore, defects in cristae organization are directly associated with a deficiency in the functionality of mitochondria [3, 4].

Outer and inner membranes separate two aqueous compartments such as the intermembrane space (IMS) and the innermost compartment referred as the matrix. IMS entails approximately a 5% of the whole mitochondrial proteome, representing the most constricted sub-compartment. However, it contains the largest variety of import-related proteins [5]. The IMS coordinates a broad range of cellular processes, including those related to respiration, metabolic support, and translocation of proteins by identifying and delivering precursor proteins to the translocase of the inner membrane (TIM) through IMS-residing hexameric complexes known as TIM chaperones [1]. They are broadly recognized for the generation of ATP, whose success mostly depends on the previous establishment of a chemical proton gradient [6]. The electron transport chain entails four multiprotein enzymatic complexes, including: (i) NADH ubiquinone oxidoreductase complex, or complex I; (ii) Succinate-Coenzyme Q reductase complex, or complex II; (iii) Coenzyme Q-cytochrome c reductase, or complex III; and (iv) cytochrome c oxidase, or complex IV. These complexes use specific substrates including NADH for complex I, while complex II employs succinate and FADH_2_ [7, 8].

Once energy-yielding reactions have finished, the IMS preserves hydrogen ions resulting from previous reactions. Therefore, hydrogen ions are employed as a free energy source for ATP synthesis. Although the conversion of ADP to ATP can occurs at the cytoplasm, most ATP molecules originate from mitochondrial reactions [7]. These processes combine the pre-established gradient of protons and their subsequent diffusion throughout the IMM in a process known as chemiosmosis [9]. Protons return to the mitochondrial matrix through an integral IMM-linked enzyme that catalyzes the addition of a third phosphate group to ADP molecules, the so-called ATP synthase, or complex V. The ATP synthase couples mechanical forces resulting from hydrogen ions diffusion with structural modifications that facilitate the addition of phosphate groups to ADP molecules resulting in ATP generation [9].

### Mitochondrial dynamics

**Fig. 1:**
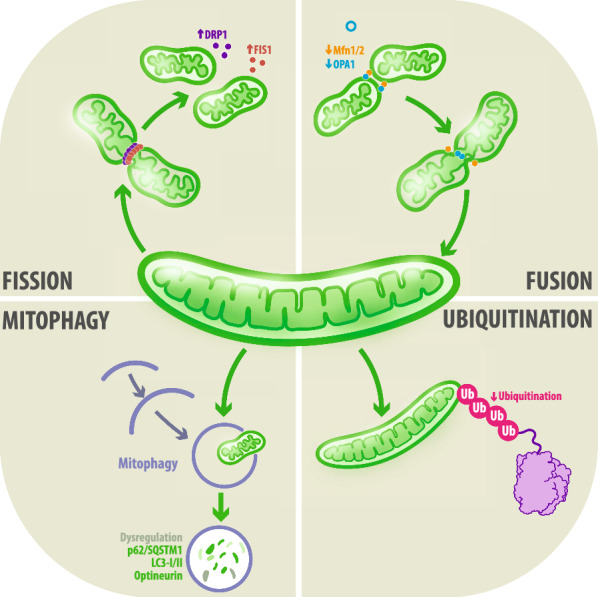
The accurate interaction between mitochondria dynamics and mitophagy: A critical bridge in health and disease. Mitochondria play a central role in cellular metabolism and function and are highly dynamic organelles that respond to environmental stimuli, activating fission, fusion, and mitophagy process. Via fission proteins, damaged mitochondria are asymmetrically divided into healthy and impaired organelle degraded via mitophagy and ubiquitin-mediated mitophagy. Moreover, the healthy fraction fuse with other mitochondria, maintain mitochondrial integrity, and regenerate the mitochondrial pool. Deregulation of these mitochondrial pathways leads to several mitochondrial dysfunctions, triggering several disorders, including neurodegenerative disease

**Fig. 2:**
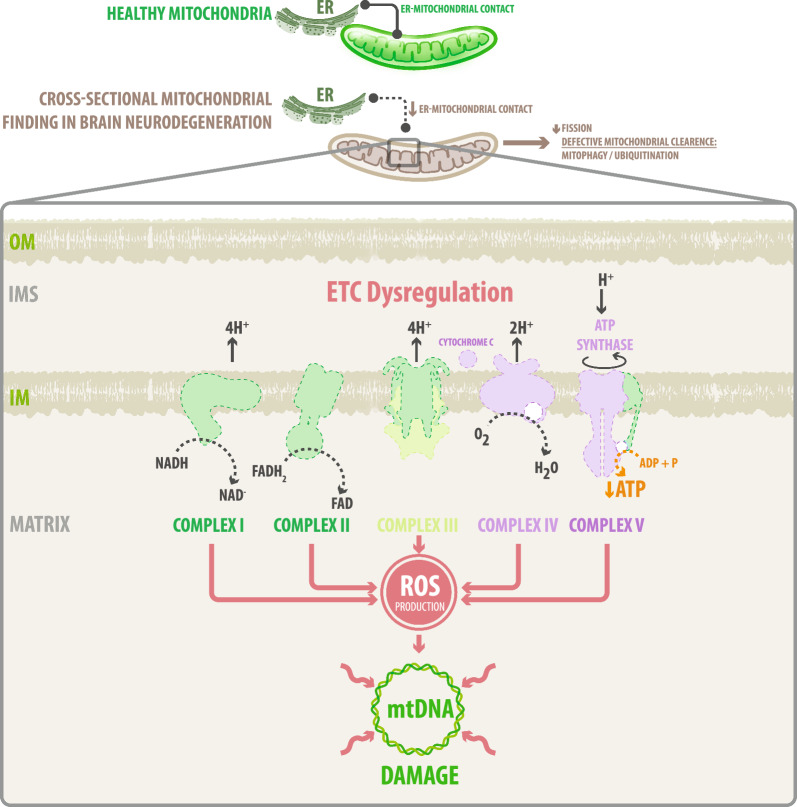
Mitochondrial dysfunction and its association with neurodegenerative disorders. Several mitochondria abnormalities are characterized by a loss of efficiency in the electron transport chain (ETC) and dysfunction in ATP-synthesis machinery. Reactive oxygen species (ROS) is a consequence of the electron transport process, produced as a by-product of oxidative phosphorylation. Furthermore, structural anomalies might lead to an increased ROS production and the release of mitochondria-derived proapoptotic factors. These free radicals can damage cellular lipids, proteins, and DNA, including mtDNA, which entails dysfunctional mitochondria associated with aging, and essentially, all chronic diseases include neurodegenerative pathologies

Mitochondria are highly dynamic organelles whose structure undergoes to coordinated cycles of fission and fusion. In turn, mitophagy is a central and selective degradation mechanism through which superfluous/damaged mitochondria are removed from the network via autophagy (Fig. 1). Abnormalities in mitochondrial dynamics are commonly observed in a wide spectrum of pathologies, including neurodegenerative disorders (Fig. 2).

#### Fission

Mitochondrial division is a multistep process that plays key roles in the quality control and spatial organization of mitochondria. It requires an extensive protein network such as the central regulators GTPase dynamin-related protein 1 (Drp-1) and mitochondrial fission 1 protein (Fis1), an inhibitor of the fusion machinery [10] (Fig. 1). Drp-1 is selectively recruited to the endoplasmic reticulum (ER)-mitochondria interface to regulate the formation of constriction sites; then, ER tubules circumscribe mitochondrial regions destined for removal, while both actin filaments aggregation and the assembly of Drp1 at ER-mitochondria interaction sites drive regional constriction and the subsequent fission [10, 11]. In turn, Fis1 acts as an inhibitor of the GTPase activity of fusion machinery proteins such as fuzzy onions/mitofusin proteins 1 and 2 (Mfn1/2) and optic atrophy 1 (OPA1) [12].

#### Fusion

Since mitochondria have double membranes, fusion events between mitochondrial units entail two separate but sequential fusion steps, which begin with the OMM and finish with the IMM [10]. The OMMs anchor to each other at the boundary through the formation and stabilization of homotypic interactions between Mfn 1 and Mfn2 and promotes membranes fusion by means of their GTPase activity [13]. On the other hand, fusion events between IMMs are mainly regulated through the mitochondrial OPA1 dynamin-like protein since their long-form mediates membranes docking, while the long and short forms conclude the coupling between already docked IMMs and, accordingly, the fusion process [10] (Summarized in Fig. 1).

#### Mitophagy

Mitophagy comprises a specific form of autophagy whose main function is the removal and recycle of superfluous/damaged mitochondria [14]. A coordinated balance between mitochondrial biogenesis and mitophagy play critical roles in the preservation of energy homeostasis [14]. Diverse stimuli can induce mitophagy through well-described intracellular pathways, including the AMP-activated protein kinase (AMPK), mammalian target of Rapamycin (mTOR) and insulin pathway, among many others. In addition, other stimuli, including nutrient deficiencies and activation of the phosphatase and tensin homolog (PTEN)-induced kinase 1 (PINK1)/Parkin pathway by hypoxia, can regulate the process of mitophagy [15].

Briefly, the recruitment and activation of mitophagy-related receptors and/or ubiquitin-autophagy adaptors lead to the formation of isolation phagophores around fragmented and non-polarized mitochondria. Afterwards, mitochondria are enclosed and sequestrated through double-membrane autophagosomes [16]. These spherical and transient structures are transported through the cytoskeleton to be fused with lysosomes where mitochondria are degraded given the flux of acidic hydrolases that favor the recycling of mitochondrial constituents [16] as observed in Fig. 1. Therefore, mitophagy-related pathways are essential to support cell survival and their deregulation is closely associated with the onset and progression of different neurodegenerative disorders. In fact, these disorders are characterized by deregulation of distinct mitophagy-related adaptors, such as p62/SQSTM1, LC3-I/II and optineurin [17, 18].

## Mitochondrial dysfunction in neurodegenerative disorders

Multiple mechanisms have been connected with the onset and progression of various neurodegenerative disorders, including: (i) deregulation of autophagy-related pathways [19], (ii) defective protein quality control [20], (iii) transcellular propagation and seeding of peptide aggregates [21, 22], (iv) synaptic toxicity and deficient network function [23], (v) abnormal stress granule dynamics [24], and (vi) mitochondrial dysfunction [25] (Fig. 2). Mitochondria play central roles in the building and functioning of brain networks, thus the breaking of mitochondrial homeostasis is repeatedly associated with progressive oxidative damage and an overall energetic failure [26]. Three major processes like fusion, fission and mitophagy form an integral part of synaptic transmission and neuronal survival. Their deregulation is correlated with degenerative mechanisms [27]. While primary mitochondrial dysfunction implies mitochondrial-autonomous defects; secondary dysfunction refers to deregulation of non-mitochondrial pathways that also entails loss of mitochondrial functioning [28] (Fig. 2). This section offers an update on mitochondrial deficiencies in diverse neurodegenerative disorders, including Alzheimer´s disease (AD), Parkinson´s disease (PD), Huntington´s disease (HD), Amyotrophic lateral sclerosis (ALS), Multiple sclerosis (MS) and Friedreich´s ataxia (FA).

### Alzheimer’s disease

AD has a prevalence ranged between 10 and 30% in the population over 65 years of age, with an estimated incidence ranging between 1 and 3% [29]. AD has a long-standing association with two major pathological hallmarks, such as accumulation of neurite tau-containing intracellular fibrillary tangles and β-amyloid (Aβ)-containing extracellular plaques derived from the amyloidogenic pathway [30]. These peptides generate progressive neuronal degeneration predominantly affecting the medial temporal lobe and neocortical structures [31], thus generating loss of cognitive skills and variable degrees of dementia. Genetic risk factors including dominant mutations in amyloid precursor protein (APP), presenilin 1 and 2 (PSEN1/2) genes [32] and dietary/nutritional factors play essential roles on AD progression [33], while age comprise the most important non-genetic risk factor [34]. Although dementia—i.e. attention, memory, reasoning, language and/or visual-spatial abnormalities—are well-recognized as a multi-etiology symptom, a growing number of studies highlights their association with mitochondrial dysfunction [25, 35].

Non-neural cell types obtained from AD patients, including fibroblasts, peripheral blood cells or platelets, present deregulation of the metabolic enzymes [36, 37]. Studies conducted by Gabuzda et al. demonstrated that the metabolic state of fibroblast-like cells act as a key determinant in the generation of amyloidogenic derivates [38], while Leuner et al. experiments support that mitochondria-derived reactive oxygen species (ROS) favor the processing of Aβ peptide (AβPP) towards Aβ in vitro and in vivo [39]. Thus, it has been hypothesized that altered mitochondrial function and energetic metabolism might precede and then favor the accumulation of tau tangles and Aβ plaques [35].

Long-standing electron microscopy findings revealed abnormal mitochondrial ultrastructure in AD brains [40, 41]. Functional experiments through fluorodeoxyglucose positron emission tomography verified that AD brains employed less glucose when compared with healthy individuals [42], while the maximal oxygen consumption estimated from brain biopsy homogenates of dementia patients showed to exceed than observed in control subjects [43].

Cybrids are cytoplasmic hybrid cells that result from combining cytoplasmic components from eukaryotic nucleated cells and non-nucleated cells (or cytoplasts) [44], and represents a useful methodology to elucidate the role of mitochondria as a causal event in degenerative diseases [45]. Experiments based on transplantation of platelets-derived cytoplasm from AD patients into human neuroblastoma SH-SY5Y non-nucleated cells induced an increase in the expression of apoptotic markers cytochrome C and caspase 3 and C terminal short tailed Aβ 1–40 variant (Aβ40) and Aβ42 [45]. These molecular changes were accompanied of reduced mitochondrial membrane potential that is restored through administration of anti-oxidant agents. AD markers such as Aβ production, apoptotic signaling, oxidative damage and mitochondrial fission showed to increase in AD cybrids, while other parameter including oxygen consumption and ATP levels exhibit a marked decrease [45].

In rats, rotenone-induced complex I inhibition generates loss of neurons in the substantia nigra and the striatum, which show cytoplasmic accumulations of 15-nm phosphorylated Tau including residues Ser^202^, Ser^214^, Ser^396^, Ser^404^, Thr^205^ and Thr^212^ [46]. Experiments performed by Escobar-Khondiker et al. support that complex I inhibition induces a decrease in the survival of embryonic rat-derived striatal neurons but also an increase in the percentage of phosphorylated Tau Ser^396^ and Ser^404^ [47]. They reported that ATP levels show a dose-dependent reduction after inhibition of mitochondrial electron transport complexes that correlates with a somatic redistribution of diverse residues of phosphorylated Tau [47], suggesting that mitochondrial bioenergetics play a central role for determining the subcellular localization of Tau protein aggregates.

On the other hand, it has been reported that transgenic mice expressing human AβPP (APP/Ld) that in turn carry a knockin mutation in mitochondrial DNA (mt-DNA) polymerase γ (PolgA D257A), show severe mitochondrial bioenergetic defects and an increase of individual´s mortality after their first year [48]. These bigenic AD mice^D257;APP/Ld^ accumulate mitochondrial DNA mutations with age, leading to elevated expression of the C-terminal long tailed Aβ 1–42 variant (Aβ42) accompanied with severe brain atrophy evidenced as poor lateral cortical thickness and hippocampal area [48]. Additionally, 6-month-old COX-deficient mice (COXd/AD) resulted from successive crosses between homozygous COX cKO mice and hemizygous mutant APP and PSEN1 transgenic mice reveal an age-dependent atrophy in the forebrain and hippocampal region, characterized by extensive neuronal loss and cortical layer disorganization [49]. COXd/AD mice show reduced expression of AβPP and COXI when compared with control AD mice, while the frequency of Aβ plaques and 8OHG^+^ oxidized nuclei were also reduced [49], tempting to speculate on the impact of mitochondrial-derived compounds in AD progression. These findings correspond with those reported by Pinto et al. whose evidence support that mt-DNA damage in COXd/AD mice reduce the number and density of diffuse Aβ plaque in the cortex and hippocampal region [50].

Mitophagy deregulation occurs normally with aging, but it turns worse in AD since that abnormal interaction between phosphorylated Tau and Aβ with mitochondria leads to suppress the expression of several mitophagy-associated proteins [51]. It has been proposed that AD-related mutations impair lysosomal maturation and thus impair the clearance of defective mitochondria [51]. This impairment generates abnormal transport of mitochondria through neuron’s body and predisposing to synaptic dysfunction. Experiments on AD patient-derived cells and mice expressing human APP show that Aβ oligomers interact with axons-residing autophagic vesicles, disturbing the expression of autophagic molecular adaptor proteins and thus leading to abnormal autophagic flux [52]. Mechanisms like release of cytochrome c from intermembrane/intercristae spaces of mitochondria, the sudden increase in Ca^2+^ intracellular concentrations, increasing oxidative stress, acquisition of mt-DNA mutations and apoptotic pathways, are well known to precede mitophagy. Thus, their long-time deregulation correlates with acquisition of mitochondrial dysfunction and subsequent neuronal death [53].

### Parkinson’s disease

PD show a prevalence nearby to 2% in the population over 50 years of age, representing the second most common brain degenerative disorders after AD [54]. PD is characterized by progressive degeneration of dopaminergic neurons in the *substantia nigra pars compacta* (SNpc), which display accumulations of Lewy bodies (LB)-containing α-synuclein protein deposits; the major pathological hallmark of PD [55]. PD-related symptoms involve motor commitment including muscle rigidity, bradykinesia and resting tremor, or non-motor symptoms, such as cognitive impairment, endocrine abnormalities, sleep dysfunction, abnormalities in pain perception and dementia, among others [54, 56]. It is widely recognized that aging represents a central risk factor [57]. However, genetic and environmental factors have been so far determined. Genome-wide associated studies mapping 18 PD-related risk loci among PARK genes (PARK1-18), including autosomal dominant mutations in α-synuclein (PARK1, 4), UCHL1 (PARK5), LRRK2 (PARK8), HTRA2 (PARK13); while recessive mutations in Parkin (PARK2), PINK1 (PARK6), DJ-1 (PARK7), and ATP13A2 (PARK9) [reviewed in [58]]. Most of these proteins were previously associated with mitochondrial dynamics [59], thus defects in mitochondrial clearance and functioning represents a frequently observed finding in PD patients. These defects have been modeled in various models, such as toxin-induced, sporadic and familial PD.

Given that, nigral neurons show a dense mitochondrial biomass with elevated ROS production, making them particularly sensitive to mitochondrial dysfunction [60]. Studies conducted by Ballard et al. prove that exposure to 1-methyl-4-phenyl-1,2,3,6-tetrahydropyridine (MTPT), a complex I inhibitor, generates permanent human parkinsonism with extensive degeneration of nigral neurons [61]. Likewise, a report on effects of pesticides employed in China, including paraquat, rotenone, fenpyroximate, tebufenpyrad and chlorpyrifos, shows that even when they are used at low doses the majority provoked mitochondrial fragmentation, ATP depletion and decreased complex I activity in SH-SY5Y cells; while pendimethalin and endosulfan generated only mitochondrial fragmentation [62]. All of them were reported for inducing a parkinsonism-like phenotype with the inhibition of ubiquitin-protease systems 26S and 20S (UPS) [62]. In fact, rats chronically treated with rotenone develop severe degeneration of dopaminergic neurons which additionally show LB-like structures [63]. MPTP-derivates such as 1-methyl-4-phenylpyridium (MPP^+^) seems to store in dopaminergic neurons causing a PD-like phenotype characterized by low mitochondrial complex I activity and poor ATP content but increased oxidative stress [64]. On the other hand, 6-hydroxydopamine (6-OHDA), a structural analogue of dopamine that carries an additional hydroxyl group linked to oxidative stress in dopaminergic neurons, provokes mitochondrial-related neuronal apoptosis in vitro [65] and motor defects in mice and rats [66]. Similarly, C. elegans worms treated with 6-OHDA present an abnormal thermal profile and increased oxidative stress as well as abnormal dopamine-dependent behavior, which seems to be closely associated with the Nrf2/Keap1 signaling pathway [65].

Most cases of PD arise sporadically showing an overall reduction in the activity of complex I in the SNpc and pre-frontal cortex [67, 68]. Complex I deficiencies are mostly associated with reduced ATP levels, excessive ROS production and calcium-induced intracellular damage [69]. These defects do not show to be connected with abnormal expression of complex I subunits [70], but rather to intrinsic issues associated with deregulation of its enzymatic properties. By comparison with cortical neurons, nigral neurons were found to develop severe mt-DNA damage in post-mortem samples from PD patients [71]; while their cerebrospinal fluid contains elevated levels of co-enzyme Q-10 and 8-hydroxy-2´-deoxyguanosine (8-OHdG), an oxidized nucleoside detected in DNA lesions [72]. As aging advances, the SNpc of PD patients progressively loses its capacity to generate mt-DNA copies, becoming susceptible to mt-DNA deletions and thus reaching up to 10% more deletions than healthy individuals [73, 74]. Myocyte enhancer factor 2D (MEF2D) functions as a transcriptional activator that binds mt-DNA sections encoding complex I subunit NADH dehydrogenase, whose down-regulation in mouse models and post-mortem PD brains is associated with increased hydrogen peroxide levels, low ATP production and stress-induced cell death [75]. Analogously, cybrid cells containing mitochondria from sporadic PD patients replicate deficiencies in complex I activity and oxidative damage, predisposing them to MPP^+^-mediated apoptosis [76].

Although familial PD comprises only a 10% of cases, Lrrk2, Parkin and PINK1 mutations have been studied in diverse models based on its association with the mitochondrial biology. LrrkG2019S knock-in mice and patient-derived fibroblasts show reduced intracellular ATP content and expression levels of mitochondrial complexes III subunit UQCR2, IV and V subunit ATP5A [77, 78]. Besides, mitochondria derived from LrrkG2019S knock-in mice stay arrested in fission [78]. Similarly, Parkin mutant models such as fibroblasts [79], Drosophila [80] and zebrafish embryos [81], exhibit reduced mitochondrial complex I activity and intracellular ATP content. In turn, PINK1 mutant zebrafish expressing a premature stop mutation (Y431*) develop dysfunction of mitochondrial complexes I and III, provoking considerable loss of dopaminergic neurons [82]. Mitochondria located nearby to synaptic terminals in the striatum of 3-months-old PINK1 knockout rats carry low complex I-mediated respiration when compared with healthy controls, and mass spectroscopy-base proteomic analyses support that, in parallel, it is accompanied by down-regulation of various electron carrier proteins [83].

### Huntington’s disease

HD is a rare autosomal dominant degenerative disease that mostly arises around 30–50 years of age. However, it can onset at earlier and more advanced stages of life [84]. HD is marked by a rapid and progressive degeneration of the cortico-striatal area of the brain but also in the globus pallidus, thalamus, subthalamic nucleus, accumbens nucleus, cerebellum and white matter [85]. Therefore, HD clinical manifestations comprise motor dysfunction, psychiatric disturbances and cognitive impairment [86]. The Htt gene encoding Huntingtin protein gains increasing number of cytosine-adenine-guanine (CAG)-repeats (> 35) in the exon 1 that is normally composed by 7–35 CAG repeats [87]. Huntingtin play critical roles in mitochondrial dynamics and functioning, regulating the expression of electron transport chain components, import of nuclear-encoded mitochondrial proteins and protection against oxidative stress [88, 89]. Deficiencies in these processes are repeatedly observed in neurons expressing mutant Htt including human samples and experimental approaches.

Although studies conducted by Hamilton et al. support that mitochondrial abnormalities are not associated with a striatal pathology [90, 91], their experiments differ with those reported by other authors. Hamilton et al. results show no differences in the respiratory activity of striatal mitochondria of the soma and neurites of YAC128 mice-derived neurons expressing human Huntingtin protein [90]. However, recent studies on inducible pluripotent stem cells (iPSC)-derived from patient-fibroblasts reveal that ATP content, respiratory and glycolytic capacities decrease proportionally to the number of CAG repeats in both neural progenitors and terminally differentiated neurons [92]; while Wistar rats exposed to increasing amounts of mitochondrial toxins develop a HD-like behavior accompanied by mitochondrial swallow, loss of cristae organization and suppression of complex III activity and ATP production in striatal cells [93]. Dysregulation of mitochondrial-encoded electron transport chain subunits has been broadly described in mutant Htt neurons (Hdh^Q111/Q111^) [94, 95], and exogenous application of glycolytic metabolites D-glucose and sodium pyruvate restores intracellular ATP content in striatal neurons [92].

Deregulation of Ca^2+^ dynamics was previously proposed as a major cause of increasing striatal vulnerability in HD. Although Hamilton et al. studies do not display significant differences in the Ca^2+^ handling capacity in non-synaptic striatal mitochondria [90, 91], experiments performed in R6/1 primary striatal neuron carrying about 145 CAG repeats exhibit reduced co-immunolocalization between GFP-Sec61β-ER and pDsRed2-labeled mitochondria, while the mitochondrial membrane potential and calcium influx are diminished, thus supporting a breaking in the ER-mitochondria interaction [96]. This is supported by experiments performed in R6/1 and Hdh^Q7/Q111^ mutant mice and patient-derived postmortem samples, which exhibit down-regulation of many mitochondrial membrane-related proteins, such as Grp75, IP3R3 and Mfn2, whose function is crucial for the maintenance of ER-mitochondria contacts in the striatum [96]. Mitochondria isolated from neuronal synaptic terminals show augmented protein expression levels of complex II 70 kDa subunit, which desensitizes them to stimuli that normally induce calcium release, such as 0.1% bovine serum albumin free from fatty acids [90].

Several studies support that mutated Htt has detrimental effects in mitochondrial fusion, fission and clearance. R6/1 primary striatal neuron develops mitochondrial fragmentation in a Drp1-dependent manner given the high number of dot-like pDs-Red2^+^ mitochondria *per* micron of microtubule associated protein 2 (MAP2)-expressing cells, which, in turn, exhibit superoxide accumulation [96]. Hdh^Q111/Q111^ neurons down-regulate the expression of fusion markers, including Mfn1/2 and OPA1, and mitochondrial biogenesis regulators such as peroxisome proliferator-activated receptor-γ coactivator 1 α and 3 β (PGC1α, PGC3β), nuclear respiratory factor 1 and 2 (Nrf1/2) and mitochondrial transcription factor A (TFAM), while fission markers Drp1 and Fis1 are significantly up-regulated [94, 95]. The relative expression of these markers is effectively restored through the treatment with antioxidants such as Mito-Q and SS31, rescuing the number of mitochondrial fragments and ATP generation, meanwhile GRPase-DRP1 activity and hydrogen peroxide levels tend to decrease [94]. Furthermore, it is reported that synaptic markers including synaptophysin, PSD95, synapsin 1 and 2, synaptobrevin 1 and 2, neurogranin, GAP43 and synaptopodin, are strongly up-regulated [94]. In turn, R6/2 HD mice show wide-ranging oxidative stress markers and reduced expression mitochondrial biogenesis mediators in both brain and skeletal muscle. These cells show high levels of autophagic markers and ubiquitination of mitochondrial constituents, which suggest that mutant Htt promotes abnormal mitochondrial degradation [97]. In fact, experiments performed by Guo et al. demonstrate that abnormal interaction between mutant Htt and valosin-containing protein (VCP) leads to mitochondrial fragmentation and excessive mitophagy in Flag-mtVCP-expressing striatal and spiny neurons, while blocking the Htt-VCP interaction outcomes beneficial for motor deficits and striatal abnormalities in both R6/2 and YAC128 mice [98].

The participation of pathogenic mechanisms including S-nitrosylation and iron storage dynamics have been previously explored [99]. BACHD transgenic rats expressing human full-length Htt (Q^97^) and postmortem brain samples obtained from HD patients show elevated levels of nitric oxide and S-nitrosylation in the striatum. Htt post-translationally modified binds with a high affinity to Drp1 and thus generate massive mitochondrial fragmentation and dendritic spine density [89]. In addition, striatal and cortical regions develop deregulation of different mitochondrial iron proteins [99]. Iron-selective chelators repair deficiencies in the mitochondrial membrane potential, oxygen consumption and ATP production in striatal and cortical neurons, while studies performed in neonatal iron supplementation showed that early exposure potentiates mitochondrial injury in R6/2 and YAC128 mice [99].

Finally, astrocytes derived from YAC128 mice and deficient in HACE1 (HECT domain and Ankyrin repeat containing E3 ubiquitin protein ligase 1) exhibit reduced Nrf2 expression, poor basal and maximal respiration and reduced ATP generation, leading to astrocytic dysfunction [88]. These mice develop accelerated onset and progression of motor abnormalities, depressive- and anxiety-like behaviors, and cognitive defects [88], emphasizing the role of astrocytes in HD.

### Amyotrophic lateral sclerosis

ALS represents a lethal disorder caused given progressive degeneration of motor neurons controlling voluntary muscles, which may eventually end in death [100]. ALS show sporadic (sALS) and hereditary (hALS) clinical presentations, however, only a 10% of cases correspond to hALS [101]. Wide-ranging mutations have been described in ALS, including superoxide dismutase 1 (SOD1) gene encoding the Cu, Zn superoxide dismutase 1 [102, 103], C9ORF72 gene encoding C9 protein [104], CHCHD10 gene encoding Coiled-Coil-Helix-Coiled-Coil-Helix Domain Containing 10 protein (C10), or protein N27C7-4 [105], TARDBP gene encoding TDP-43 protein [106], FUS gene encoding RNA binding protein Fused in Sarcoma (FUS), and OPTN gene encoding optineurin protein [107], among others. Mitochondrial dysfunction represents a common finding in all models that carry these mutations [108, 109]; while cross-sectional clinic radiological studies based on ^31^P-magnetic resonance spectroscopy in ALS patients revealed that brain motor areas and the tibialis anterior portion exhibit primary dysfunction of mitochondrial oxidative phosphorylation [109]. For a more detailed description on both molecular and cellular findings connecting ALS-related mutations and mitochondrial dysfunction, the reader is redirected to Table 1.

**Table 1:** Mitochondrial deficiencies associated with brain degenerative disorders

Disease	Molecular Hallmarks		Cellular Hallmarks	Model		References
Alzheimer's disease (AD)	n.d		Metabolic enzyme dysregulation, favoring the accumulation of tau tangles and Aβ plaques	Non-neural cell types obtained from AD patients		[35–37]
	Increased expression of apoptotic markers		Reduced mitochondrial membrane potential, decreased ATP levels and oxygen consumption	Human neuroblastom a SH-SY5Y non-nucleated cells transplanted with platelet- derived cytoplasm from AD patients		[45]
	Rotenone- induced complex I inhibition		Loss of neurons in the substantia nigra and the striatum, cytoplasmic accumulations of phosphorylated tau. Decrease in ATP levels	Rat		[46, 47]
	Accumulating mitochondrial DNA polymerase mutation		Severe mitochondrial bioenergetic defects	Transgenic mice expressing human AßPP		[48]
	Reduced expression of AßPP and COX1		Forebrain and hippocampal region atrophy. Reduced Aß plaque frequency and reduction of 8OHG^+^ nuclei	COX deficient mice		[49]
Parkinson's disease (PD)	Complex 1 inhibition		Nigral neuron degeneration	SH-SY5Y cells Rats treated with rotenone		[62, 63]
	Nrf2/Keap1 signaling		Abnormal thermal profile, increased	Mice, rats, and C. elegans		[65, 66]
	Pathway		Oxidative stress, and abnormal dopamine dependent behavior. Mitochondrial related neuronal apoptosis and motor defects	Treated with 6- OHDA		
	Severe mt-DNA damage		Elevated levels of co-enzyme Q-10 and 8-OHdG on nigral neurons	Post-mortem samples from PD patients		[71, 72]
	MEF2D binding to mt-DNA sections encoding complex I subunit NADH dehydrogenase		Increased hydrogen peroxide levels, low ATP production and stress-induced cell death	Mice and post mortem PD brains		[75, 76]
	Expression levels of mitochondrial complexes III subunit UQCR2, IV and V subunit ATP5A		Reduced intracellular ATP content	LrrkG2019S knock-in mice and patient- derived fibroblasts		[77]
	Parkin mutation		Reduced mitochondrial complex I activity and intracellular ATP content	Human fibroblasts, drosophila and zebrafish		[79–81]
	Premature stop mutation (Y431*)		Dysfunction of mitochondrial complexes I and III, and loss of dopaminergic neurons	PINK1 mutant zebrafish		[81]
	Down-regulation of various electron carrier proteins		Low complex I- mediated respiration	PINK1 KO rats		[83]
Huntington's disease (HD)	Decrease in CAG repeats		Decrease in ATP content, and respiratory and glycolytic capacities	iPSC-derived from patient fibroblasts		[92]
	Dysregulation of mitochondrial encoded genes associated with electron transport chain subunits		Mitochondrial swallow, loss of cristae organization, suppression of complex III activity and ATP production	Wistar rats exposed to mitochondrial toxins		[93–95]
	Approximately 145 CAG repeats, downregulation of mitochondrial membrane related proteins		Mitochondrial membrane potential and calcium influx are diminished	R6/1 primary striatal neuron		[96]
	Down-regulation of the expression of fusion markers and mitochondrial biogenesis regulators Upregulation of fission markers		Decrease in mitochondrial fragments and ATP generation. Increase in GRPase-DRP1 activity and hydrogen peroxide levels	HdhQ111/Q11 1 neurons		[94, 95]
	Increased oxidative stress markers and reduced expression of mitochondrial biogenesis mediators		Increase in autophagy and ubiquitination of mitochondrial constituents	R6/2 HD mice		[97]
	Mutant Htt interacting with VCP		Mitochondrial fragmentation and increase in mitophagy	Flag-mtVCP- expressing striatal and spiny neurons from R6/2 and YAC128 mice		[98]
	Transgenic expression of human full length Htt (Q^97^). Htt binding with Drp1		Increase in nitric oxide and S- nitrosylation. Promotion of mitochondrial fragmentation and dendritic spine density	BACHD transgenic rats and postmortem brain samples from HD patients		[89]
Multiple	Disturbed		Calcium	Brain tissue		[122]
Sclerosis (MS)	Oxidative phosphorylation, activation of calcium- dependent proteases		Accumulation, cytoskeletal modifications, and impaired axonal integrity	From patients autopsy		
	Dysregulation of the expression of H3K3me3		Change in chromatin dynamics and thus poor transcription of mitochondrial genes	Human SH- SY-5Y neuroblastom a cells		[127]
	Sirtuin deregulation and Rab32- mediated ER stress		Mitochondrial dysfunction and thus progressive neuronal death and MS severity	Primary human fetal neurons		[116]
	n.d		Low respiratory capacity, poor mitochondrial mass and reduced proliferative potential	Peripheral blood cells of MS patients		[114]
	Down-regulation of mitochondrial biomass markers		Ultrastructural mitochondrial abnormalities	Naïve and effector memory CD4^+^ T cells from MS patients		[114]
Friedreich's ataxia (FA)	Frataxin knock- down, up- regulation of SOD1/2 enzymes		Disturbances in mitochondrial membrane potential and dynamics, elevated protein carbonylation and poor reductive capacity, increased lipid droplet formation	Non-neural cells		[138, 139]
	n.d		Glutathione- dependent mitochondrial oxidative stress and thiol modifications in respiratory chain	Frataxin deficient lymphoblasts		[140, 141]
			complexes III and IV			
	Depletion of frataxin expression		Poor neuronal cell area in the dorsal root ganglia	Frataxin- deficient knockin- knockout (Cg- Fxntm1MknFx ntm1Pand/J; KIKO) mice		[144]
	Single GAA repeat sequence in the frataxin gene		Reduced mitochondrial membrane potential, exacerbated ROS generation, defects in the activity of mitochondrial complexes I, II, III and IV, and increased lipid peroxidation and neuronal death	YG8R mice		[146]

n.d.: non-described

### Multiple sclerosis

MS comprises an autoimmune and neurodegenerative disorder caused by progressive demyelination and axonal dysfunction, generating a wide-ranging number of symptoms from visual and sensory defects to ataxia and sexual dysfunction, weakness, pain syndromes, depression, and cognitive damage [110]. MS display distinct forms including: (i) clinically isolated syndrome, which can be considered a single MS-like episode; (ii) relapsing–remitting MS, including presentation of neurological symptoms separated by remission times; (iii) secondary progressive MS, regardless of remission and presenting a broader spectrum of neurological symptoms; and (iv) primary progressive MS, which may comprise lesions at the brain and/or spinal without remission periods and becoming progressively worse once MS begins [111]. Distinct cellular constituents participate in the onset and progression of MS such as neurons, oligodendrocytes (OLs), astrocytes, B cells, CD4^+^ and CD8^+^ T cells, and microglial cells [112–115]. All of them have exhibited mitochondrial defects in MS models, containing defects in redox and iron homeostasis, ER-induced irregular mitochondrial structure and/or energetic dysfunction [113, 116, 117]. Besides, there is a clinical overlap between MS and other primary mitochondrial disorders such as Leber hereditary optic neuropathy, Leigh syndrome, mitochondrial encephalopathy, myoclonic epilepsy with ragged-red fiber and optic atrophy type 1 [110].

Myelinated axons concentrate mitochondria mostly within the juxtaparanodal and internodal axoplasm [118, 119]. Aggressive MS subtypes exhibit down-regulation of mt-DNA-encoded complex IV catalytic subunit at lesion sites [120]. Axonal tracts in MS lesions in both the brain and spinal cord exhibit glutamate-mediated dysfunction of complex IV [119]. Since exists an inverse correlation between complex IV activity and the density of microglial cells at lesion sites [119], it has been proposed that these cells are able to perpetuate a state mitochondrial dysfunction. Mitochondria migrate at demyelinated sites across axons as a compensatory mechanism to promote remyelination via Miro1 and PGC-1α/PPARγ pathway. Nevertheless, the chronic activation of the immune system causes changes in the number, size, functionality and migration of neuron-residing mitochondria, which leads to the impairment of this healing mechanism [118]. In turn, the blockade of mitochondrial biogenesis shows to increase the frequency of dorsal root ganglia neurons lacking myelin markers, favoring the establishment of demyelinated tracts and thus the deterioration of axonal dysfunction [121].

Neurons carrying oxidative phosphorylation disturbances exhibit calcium accumulation, the activation of calcium-dependent proteases and thus cytoskeletal modifications that impair axonal integrity [122]; while OLs are particularly sensitive to oxidative stress, showing selective loss at demyelinated lesions as it promotes glutamate-induced toxicity [123, 124]. Recent studies reveal that defects in mitochondria-related pathways are fundamental determinants in the survival and differentiation of OLs both in vitro and in vivo [125]. Given that activation of ionotropic glutamate receptors provoke calcium-dependent mitochondrial toxicity [126], it is expected that the frequency and functionality of OLs remains altered as demyelination progress. In turn, the methyl donor betaine was recently described given that its overall down-regulation in MS brain tissue deregulates the expression of histone H3 trimethylated on lysine 4 (H3K4me3), whose impacts on chromatin dynamics leads to poor transcription of mitochondrial genes [127]. Notably, the rescue of mitochondrial respiration correlates with an enhancement of sensorimotor disabilities in models of toxic demyelination [127]. Other alternative mechanisms, such as sirtuins deregulation and Rab32-mediated ER stress, demonstrate that resulting mitochondrial dysfunction acts as a triggering event in progressive neuronal death and MS severity [116, 128]. Therefore, MS-related axonal degeneration following structural and functional deficiencies in mitochondria may be understood as a multistep process.

In MS-patients, inflammatory lesions in the central nervous system (CNS) contain CD4^+^ and CD8^+^ T cells, while B cells and other immune cells are structured as ectopic germinal bundles in the meninges [129]. There is a tight balance in the activity of pro-inflammatory populations including B cells, CNS dendritic cells, CD4^+^ and CD8^+^ T cells and M1 macrophages; and anti-inflammatory cells, such as M2 macrophages and CD4^+^ regulatory T cells (Treg) [130]. Peripheral blood of MS-patients shows increased number of pro-inflammatory cell populations, including CD8^+^, Th1 and Th17 cells. These cells have a low respiratory capacity and poor mitochondrial mass, while they are mostly terminally differentiated/exhausted cells with a reduced proliferative potential [114]. CD3/CD28-mediated activation of CD4^+^ naïve T cells obtained from patients carrying primary progressive MS, exhibit low basal and maximal respiration capacity and poor ATP production [114]. Besides, naïve and effector memory CD4^+^ T cells develop a more aggressive MS phenotype characterized by down-regulation of mitochondrial biomass markers and ultrastructural mitochondrial abnormalities [114]; while peripheral blood nuclear cells (PBMCs) obtained from MS patients display low reductive activity and increased ROS generation [112]. In turn, experiments conducted in an experimental autoimmune encephalomyelitis (EAE) a murine model for study MS, reveal that mitochondria-targeted antioxidants are efficient to reduce the frequency of Iba1^+^ and IL-6^+^ cells, likely corresponding to M1 macrophages and pro-inflammatory cells [131]. Furthermore, the authors show that antioxidant usage ameliorates neurotoxicity induced by LPS-activate microglial cells in spinal cord lesions and restore the clinical score of EAE mice [131]. Functional polarization of monocytes/macrophages and/or lymphocytic phenotypes play crucial roles in the regulation of inflammatory processes, thus representing key targets to prevent the degeneration of diverse systems, including the CNS [132, 133].

### Friedreich’s ataxia

Friedreich’s ataxia (FA) is a rare autosomal recessive and progressive neurodegenerative disorder resulting from atrophy and loss of neurons in the dorsal root ganglia, spinal cord, dentate nuclei, brainstem and cerebellum, leading to deficiencies in verbal and cognitive skills [134, 135]. However, it also affects non-neural tissues like heart and skeletal muscle, among others [136]. FA outcomes from a genetic mutation on the frataxin gene, thus producing a trinucleotide guanine adenine-adenine (GAA) triplet repeat expansion on its first intron that generates a hypomorphism at the protein level. Frataxin is a small mitochondrial protein involved in the formation of iron-sulphur (Fe/S) clusters which represent essential co-factors in the transport of electrons through respiratory chain complexes [136]. Therefore, the molecular and cellular mechanisms involved in FA pathogenesis entail structural and functional abnormalities in mitochondria.

By analogy with murine models, the mature frataxin form represents only a 7 to 15% of total frataxin, while the rest corresponds to a truncated proteoform lacking the N-terminal lysine residues [137]. In non-neural cells, frataxin knock-down generates disturbances in mitochondrial membrane potential and dynamics, elevated protein carbonylation and poor reductive capacity. These events correlate with up-regulation of SOD1/2 enzymes and medium-chain acyl-CoA dehydrogenase levels and in turn augmented lipid droplets formation. These findings give rise to a FA-associated cardiomyopathy phenotype, cardiac stenosis and then hearth failure [138, 139]. Proteomic and imaging studies reveal that lymphoblasts carrying frataxin-deficiency develop severe glutathione-dependent mitochondrial oxidative stress accompanied by thiol modifications in respiratory chain complexes III and IV [140, 141], while proximity ligation assays support that ER-mitochondria contacts and calcium uptake dynamics are deregulated [142].

Frataxin expression acts as a protective mechanism against ROS generation and supports mitochondrial functionality through restoring antioxidant enzymes levels and mitochondrial complexes activity [143]. FA patient iPSC-derived sensory neurons show extensive oxidative stress and reduced basal and maximal respiration capacity [144], while frataxin-deficient knockin-knockout (Cg-Fxn^tm1Mkn^Fxn^tm1Pand^/J; KIKO) mice exhibit poor neuronal cell area in the dorsal root ganglia, which is rescued through restoring frataxin expression [144]. Furthermore, progressive accumulation of inorganic iron deposits in the mitochondrion promotes serious oxidative damage in different brain compartments, low ATP content and neurodegeneration [145]. In turn, cerebellum-derived granule and glial cells from YG8R mice, which carry a single GAA repeat sequence in the frataxin gene, show reduced mitochondrial membrane potential, exacerbated ROS generation, defects in the activity of mitochondrial complexes I, II, III and IV, increased lipid peroxidation and neuronal death [146]. Additionally, experiments performed by La Rosa et al. support that FA-related oxidative damage may be also associated to deregulation of the NRF2 signaling pathway; a major regulator of the antioxidant response [147].

**Fig. 3:**
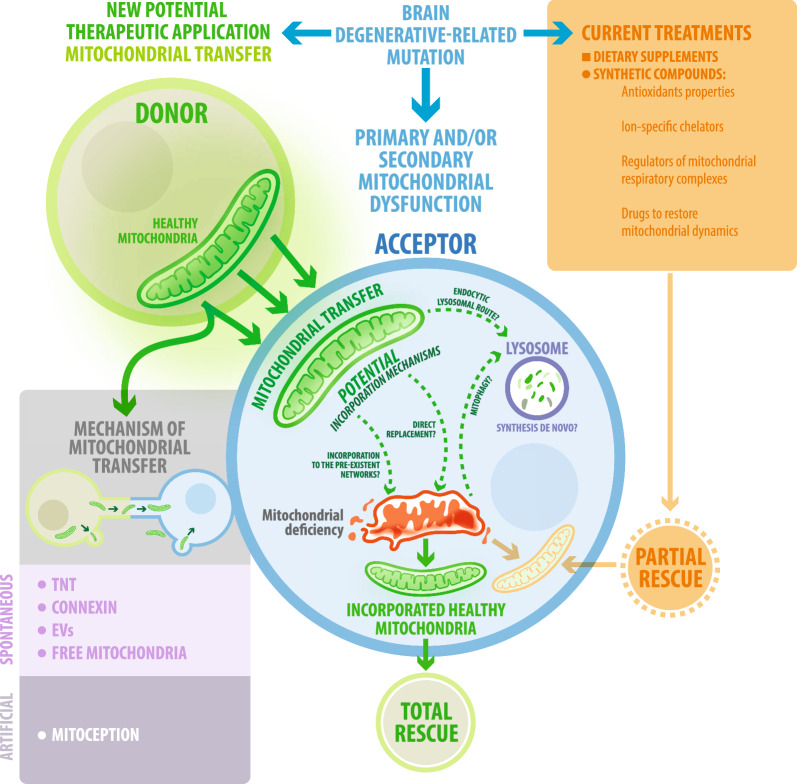
Mitochondrial transfer as a new therapeutic strategy to treat neurodegenerative diseases. Therapies that target mitochondria have emerged as a promising strategy for reducing oxidative stress and restoring mitochondrial function. However, limitations in specificity and drug delivery have hindered the success of these current treatments. Therefore, novel therapeutic approaches are critically needed. One of these is mitochondrial transfer. Mitochondria can be transferred from one cell to another; this process involves spontaneous deliveries such as tunneling nanotubes (TNT), extracellular vesicles (EVs), gap junctions, but also artificially through mitoception. All of these mechanisms allow the transfer of intact and functional mitochondria, increasing the mtDNA content of the acceptor cells and thus restoring their capacity for respiration and survival. In the neurodegenerative disease’s context, artificial and/or spontaneous mitochondrial transfer might induce a total rescue of neurodegenerative acceptor cells through several unknown mechanisms that are still under investigation

All this brain degenerative disease expresses diverse forms of mitochondrial dysfunction, which are summarized in Table 1. Deficiencies in the functionality of mitochondria increases ROS production and provoke mt-DNA damage as represented in Fig. 2. Therefore, the generation of novel strategies aimed to reverse the state of mitochondrial dysfunction might prevent the decline of nervous cells and thus ameliorate brain degeneration (Fig. 3).

## Mitochondrial transfer as a promising therapeutic strategy for brain degeneration

Most developing therapies aimed at treating neurodegenerative disorders have centered on restoring the functionality of mitochondria. These agents include dietary supplements and synthetic compounds with antioxidant properties, ion-specific chelators, regulators of mitochondrial respiratory complexes, and drugs aimed at restoring mitochondrial dynamics [148–150]. In this regard, we recently discussed traditional therapeutic approaches for restoring mitochondrial dysfunction, including minocycline, polyphenols, vitamins like A, D, E and B3, co-enzyme Q10, creatine, mitoquinone and monoamine oxidase B inhibitors [151]. Indeed, both safety and cost-effectiveness show a positive balance. Still, none of these methods can comprehensively address all defects, while de novo-generated mitochondria are expected to be conditioned by the pathological microenvironment.

In the last year, a new strategy based in the transplantation of healthy mitochondria to restore mitochondria dysfunction has been proposed. Long-standing evidence supports that mitochondria can be horizontally transferred from a donor cell to another both in vitro and in vivo [152–155]. A growing number of studies reveal the benefits of mitochondrial transfer in recipient cells, including an improvement on their metabolism and function, once they acquire mitochondrial units through spontaneous and/or artificial transfer [152–155]. For neurodegeneration, mitochondrial transfer offers the acquisition of exogenous mitochondria as a “fresh” source that lacks the pathological background of these disorders. Therefore, both molecular and cellular abnormalities could be restored together and not as separate events. The following section reviews the so far described types of mitochondrial transfer and up-to-date mechanisms at the molecular and cellular levels. We highlight how reiterative is mitochondrial transfer between different cell types that participate in central and/or peripheral nervous degeneration, and how beneficial is mitochondrial transplantation in nervous tissues when exposed to acute or chronic impairment (Fig. 3).

### Spontaneous mitochondrial transfer

Naturally occurring mitochondrial transfer has been reported in different cell types through mechanisms including the release of extracellular vesicles (EVs) [156], the formation of transitory tunneling nanotubes (TNTs) structures [154, 157], the opening of inter-cellular Gap junctions [152], and the release of non-cover mitochondria towards the extracellular medium [158], as represented in Fig. 3.

EVs are lipid bound carriers that cells secreted to the extracellular space mostly in three subtypes: microvesicles, exosomes and apoptotic bodies [159]. Two widely accepted criteria to discriminate between EVs include their size and content, comprised by lipids, nucleic acids, and plasma membrane and cytosolic proteins [159]. In this regard, microvesicles ranged between 500 and 900 nm function as vehicles for structurally and functionally intact mitochondria, whose production and subsequent release is regulated through the CD38/cADPR signaling and downstream Ca^2+^-dependent mechanisms [156]. The incorporation of mitochondria seems to occur in an integrin-mediated Src/Syk-dependent manner, giving rise to an energetic balance in recipient cells [156]. On the other hand, exosomes ranged between 50 and 100 nm, carry mitochondrial components, including compartment-specific proteins and electron transport chain complexes. Segments of mitochondrial units are structurally and functionally intact, enabling them to incorporate into the host network [160].

On the other hand, TNT are produced by means of filopodia-like cell membrane protrusions through which cells extend to fuse with each other, supporting the formation of narrow and substrate-independent bridges for trafficking distinct cellular constituents, including mitochondria [161, 162]. These structures are typically conformed by actin filaments and perform several functions including the rescue from energetic damage, the regulation of mechanisms involved in cellular differentiation and plasticity, and the modulation of the immune response [155, 162, 163]. Miro1, a Rho GTPase, regulates the formation of actin-based intercellular structures, and its pharmacological and genetic suppression correlates with a lower rate of mitochondrial transfer and therefore loss of metabolic and functional benefits in neural stem cells (NSC) and primary cortical neurons [154, 157].

Gap junctions comprise specialized intercellular channels between adjacent cells, that favor the rapid exchange of small molecules and participate in a broad variety of physiological processes in every tissue and system, including the nervous system [164]. Conformed essentially by connexins, the function of Gap junctions is critical during mitochondrial transfer; studies support that their pharmacological modulation directly influences on the integration of mitochondrial units in host cells, while benefits in the respiratory capacity and ATP synthesis display a rapid decline when Gap junctions are inhibited [152]. Remarkably, experiments performed in rats’ spinal cord injury suggest that cell-free mitochondrial transplantation promotes up-regulation of the Gap junctions’ constituent Connexin-43 [152], representing a novel target to explore the modulation of mitochondrial transfer both in vitro and in vivo.

Lastly, a growing number of studies support that cells can release naked mitochondria, or isolated mitochondrial constituents such as functional respiratory complexes and nucleic acids. Cell-free circulating mitochondrial DNA and whole mitochondrial units are implicated in the regulation of inflammation processes once cells extrude them [165, 166], showing a high efficiency because their independency from receptors and coupled proteins. Even cell-free mitochondria and/or constituents have been previously discussed because their relevance in people aging and obesity [166]. Thus, artificial mitochondrial isolation and following transplantation in recipient cells are employed as an experimental approach aimed to replicate the naturally occurring transfer.

### Artificial mitochondrial transfer

The symbiotic origin of mitochondria and spontaneous mitochondrial transfer toward damaged cells, are central features for the development of novel therapeutic approaches [162, 167]. Mitoception (MC) refers to the artificial internalization of mitochondria derived from a donor cell type to recipient cells, utilizing co-incubation and centrifugation approaches that favor their integration in vitro [167]. Although MC was recently coined by Caicedo et al. in 2015 [167], long-standing evidence from early 80’s support the biological benefits of the artificial mitochondrial transfer [162].

MC differs from classical co-incubation methods given the incorporation of two additional steps: centrifugation and a thermic shock. These variations favor the constant uptake of mitochondria, whose incorporation rate is proportional to the amount of mitochondrial mass disposed when co-incubating [167]. As occurs with spontaneous mitochondrial transfer, MC can restore several metabolic parameters in the recipient cells including basal and maximal respiration, mitochondrial membrane potential and biomass, and ATP content. Their improvements on functional aspects have been described in neurodegeneration and cancer models, involving invasive potential, plasticity, survival, proliferation, specific lineage commitment and differentiation in recipient cells [155, 167, 168].

### Internalization of cell-free mitochondria

Different mechanisms have been proposed to explain the internalization of mitochondria, including endocytosis mediated by caveolae/clathrin and actin and macropinocytosis [169–172]. Advanced imaging approaches support that internalized mitochondria are transported to endosomes and lysosomes, but most can escape the endolysosomal route to fuse directly with the endogenous recipient network [173]. Thus, it is expected that the uptake of mitochondria occurs through different pathways.

### Mitochondrial transfer towards central and peripheral-residing nervous cells

Current evidence support that nervous cells including diverse types of neurons [152, 174, 175], astrocytes [156, 174], neural and endothelial progenitors cells [176], as well as macrophages [175] and T cells [155, 163], can act as donor and/or host cells for mitochondrial transfer. As earlier mentioned, most of these cell types participate in neurodegenerative disorders, which, in turn, correlate with different mitochondrial abnormalities at the structural and functional level. On the other hand, further types of multipotent stem cells including mesenchymal stem/stromal cells (MSC), which are rarely found in nervous tissues, are widely recognized because their broad regenerative potential and ability to transfer mitochondria [153, 154, 157, 163, 177].

The impact of mitochondrial transfer from astrocytes to neurons have been documented in different models of stroke, toxic-induced cognitive impairment, and Alexander disease-related to GFAP mutations [156, 174, 178]. Approximately a 53% of astrocyte-derived extracellular particles oscillating between 500 and 900 nm transport structurally and functionally healthy mitochondria, being released in a Ca^2+^-dependent manner through the regulation of the CD38/cADPR signaling [156, 178]. These mitochondria are dynamically transferred into MAP2^+^ neurons through a mechanism involving the integrin-mediated src/syk axis, thus increasing their intracellular oxygen consumption and ATP levels, and conferring neuroprotection against cerebral ischemia [156]. Comparable observations were performed by English et al. whose experiments on cisplatin-treated neurons suggest that astrocytes can sense the impairment of neuronal mitochondria. Cisplatin-treated neurons acquire 35% more mitochondria than healthy neurons, and receptor neurons exhibit an improvement on their survival, mitochondrial membrane potential and calcium homeostasis, in a Miro1-dependent manner [174]. Reciprocally, up to 5% of cultured astrocytes integrate mitochondrial units from donor neurons, which is inhibited through genetic and pharmacological ablation of Miro1 and CD38/cADPR signaling pathways [178]. Besides, it has been described that a 5.36% of astrocytes are capable of transferring mitochondria to each other, while those carrying GFAP mutations lose that ability [178]. This is attractive since Alexander´s mutation is pathologically associated to dystrophic astrocytes containing intermediate filaments aggregates, poor survival and thus different myelin defects [179].

Brain endothelial cells, pericytes/pericyte-like cells and/or endothelial progenitor cells (EPC), exhibit an intrinsic ability to secrete mitochondrial units to the extracellular medium [176]. EPC are capable of transferring up to 13% of their whole mitochondrial network to the extracellular media, showing pro-angiogenic effects, modifying the endothelial tightness through the stabilization/integration of cadherins and occludin at anchoring points between endothelial cells, and restoring brain endothelial energetics and integrity after oxygen–glucose deprivation [176]. On the other hand, experiments recently performed by Van der Vlist et al. highlight the impact of macrophages-mediated mitochondrial transfer on the establishment and resolution of inflammatory pain. In this regard, CD206^+^ macrophages are capable to migrate and accumulate in the dorsal root ganglia in order to transfer their mitochondria to sensory neurons previously treated with carrageenan [175]. Approximately a 4.5% of sensory neurons incorporate Mito-Dendra signal from macrophages obtained from LysM^cre^-Mito-Dendra2^flox^ mice, whose efficiency attenuates the pain in a mouse model of carrageenan-induced hyperalgesia [175].

Adult NSC are predominantly confined to the dentate gyrus located in the hippocampus and the subventricular zone of the lateral ventricles, representing a self-repairing mechanism that generates functional neurons after brain injury [180]. Proteomic and gene ontology analyses reveal that NSC-derived extracellular vesicles transport three major mitochondrial constituents and compartments, including outer and inner membranes and matrix as well as subunits of the five mitochondrial complexes expressed from mitochondrial and nuclear genomes [160]. These mitochondria are structurally and functionally healthy as determined through electron transmission microscopy and high-resolution respirometry, and their incorporation in the network of pro-inflammatory mononuclear phagocytes blocks their metabolic switch [160]. Likewise, experiments performed in EAE, highlights that NSC transplantation is mostly distributed and incorporated by F4/80^+^ mononuclear phagocytes and GFAP^+^ astrocytes, whereas T cells, neurons and oligodendrocytes do not integrate them [160]. These variations in the faculty to internalize mitochondria might be explained through different ways. Although astrocytes are predominantly glycolytic, their capacity to incorporate mitochondria is closely associated with their role in mediating the transcellular degradation of mitochondria from neighboring cells. This process is known as transmitophagy and represents a fundamental mechanism for the maintenance of the energetic homeostasis in neurons [181]. On the other hand, the metabolism of nervous and peripheral phagocytic cells is associated with their plasticity to polarize into M1 or M2-like phenotype. Thus, it is expected that in an inflammatory context where pro-inflammatory M1 cells are predominant, there may be a tendency to incorporate mitochondria since their respiration is altered [133, 182]. The increase in OXPHOS metabolism correlates with reprogramming M1-like cells towards M2-like cells [133]. An elevated incorporation of mitochondria may reflect the transition of M1-like cells toward an anti-inflammatory phenotype. Furthermore, macrophages play critical roles in pain resolution by transferring mitochondria to sensory neurons. Furthermore, macrophages play critical roles in pain resolution employing the transfer of mitochondria to sensory neurons [175]. Although these physiological roles of astrocytes and phagocytes may explain the differences in the internalization of mitochondria depending on cell type, these interpretations still need to be corroborated.

Although MSC are not commonly found in nervous tissue, their high regenerative potential, capacity to transdifferentiate to neural cells in vitro and ability to transfer mitochondrial to most of cells types involved in brain degeneration, have generated increasing attention [155, 163, 177, 183]. Mitochondrial transfer from MSC have been reported toward (i) CD4^+^ and CD8^+^ T cells [155, 163, 177], (ii) spinal cord-residing motor neurons [152], (iii) cortical neurons [154], and (iv) hippocampal and subventricular adult NSC [157]. Experiments performed by our laboratory and others support that human Th17 cells incorporate mitochondria from bone marrow-derived MSC (BM-MSC) under co-culture conditions, showing a reduction in the overall production of the pro-inflammatory cytokine IL-17, an increase in their maximal respiration and favoring their transition toward Treg cells [155, 163]. Mitochondrial transfer from MSC to T cells seems to occur through TNT formation in a CD93/CD73-dependent manner, and its blockade stops their transition toward Treg cells [177]. MSC-derived mitochondria promote changes in mRNA expression associated with T cell activation and differentiation pathways, and enhanced immunosuppressive potential associated with increased frequency in the population of CD127^low^CD25^+^FoxP3^+^ Treg cells [155]. Thus, resulting Treg modulate the proliferative activity of human PBMCs, while experiments aimed to inject mitocepted PBMCs in a xenogenic model of Graft versus Host Disease (GvHD) improve the resolution of the inflammatory process and survival of individuals [155]. Additionally, experiments carried out by Do et al. support that MSC promote an increase in the frequency of Treg but also suppress the population of exhausted and pro-inflammatory T cells [177], while Treg that accepted mitochondria also suppress inflammation even more efficiently than control Treg [177]. By comparison, BM-MSC-derived mitochondria from patients diagnosed with rheumatoid arthritis cannot replicate these outcomes, and T cells display a poor incorporation of mitochondria when compared with those isolated from healthy MSC [163].

On the other hand, a recent study determined the impact of MSC-derived mitochondria on the locomotor functionality after spinal cord injury [152]. BM-MSC can transfer mitochondria to ventral spinal cord motor neurons through multiple experimental approaches including cell-to-cell contact, conditionate medium and artificially isolated mitochondria. These mitochondria increase the neuronal ATP content, mitochondrial membrane potential as well as basal and maximal respiration [152]. These variations correlate with reduced early and late apoptosis after oxygen–glucose deprivation in vitro*,* while motor neurons in the ventral horn of rats are enriched in BM-MSC-derived mitochondria after injury. In fact, 6-week after mitochondrial transplantation there is a significant increase in locomotor function, which correlates with an increase in the myelin content and reduction in collagen and cavity areas [152]. Interestingly, retinoid acid and 18beta-glycyrrhetinic acid (18β-GA), a potentiator and inhibitor of gap junction’s functionality, respectively, modify the integration dynamics and beneficial effects of BM-MSC-derived mitochondria on motor neurons, complementing the TNT formation as an alternative mode of transferring mitochondria through cell-to-cell contact [152].

Finally, MSC-mediated mitochondrial transfer has also been proved for being beneficial in stroke and exposure to neurotoxic agents [154, 157]. Imaging and flow cytometry experiments demonstrate that primary cortical neurons exposed to oxidative damage via hydrogen peroxide internalizes more MSC-derived mitochondria than healthy controls, helping to repair their basal/maximal respiration and ATP content [154]. These effects seem to be mediated through the formation of TNT given the up-regulation of TNFAIP2 in a Miro1-dependent manner [154]. Similar observations were performed in a model of cisplatin-induced neurotoxic effects. In this regard, NSC exposed to 0.5–1 µM cisplatin become susceptible for cell death, showing poor oxygen consumption and ATP content in vitro, which are efficiently restored through co-culture with MSC [157]. Mitochondrial transfer from MSC toward NSC is regulated via Miro1 involving the formation of actin-based intercellular structures, whose inhibition via 2 µM Latrunculin B impacts on the transfer effectiveness and its benefits in NSC [157]. On the other hand, experiments performed in 9 weeks-old mice exposed to the administration of cisplatin (2.3 mg/kg/day) reveal a reduction in the number of DCX^+^ neural progenitors in the hippocampus and lateral ventricles, which is accompanied by mitochondrial dysfunction. These alterations are efficiently recovered in vitro, while nasal administration of MSC suspensions (1 × 10^6^ cells per mouse per day) restore the neurogenic processes in the hippocampus and lateral ventricles [157].

Altogether, these data highlight the potentiality of MSC-mediated mitochondrial transfer for restoring the functionality of mitochondria in multiple cell types, and to induce tissue revitalization in the nervous system, even when mitochondrial dysfunction is stablished.

## Exploring mitochondrial transfer as a potential therapeutic approach for neurodegenerative disorders: From pre-clinical models to clinical translation

Pioneer studies by McCully et al. have called increasing attention since transplantation (injection) of healthy mitochondria on ischemic lesions exhibit pro-regenerative effects in pre-clinical animal models and human patients. Although most research has focused on myocardial ischemia and reperfusion injury [184–187]; biodistribution and stereotactic studies show that mitochondria can be efficiently incorporated in the brain parenchyma after middle cerebral artery occlusion and ischemia [168, 188]. Besides, the pro-regenerative effects of intravenous injection of mitochondria were recently studied in neurodegeneration [168].

Both epicardial and coronally injected mitochondria are capable to displace within the ischemic tissue. Once there, authors noted ameliorated oxidative stress accompanied by an overall reduction in the magnitude of infarct foci and myocardial cell death, while the generation of ATP and ventricular dynamics showed a robust correction [184–186]. Studies conducted in a porcine model show that transplanted mitochondria can reduce up to an 8% the infarct size [189]. Analogously, a pilot clinical study performed on five pediatric patients carrying myocardial ischemia and demanding extracorporeal membrane oxygenation revealed that mitochondria transplantation promote an enhancement in the ventricular function associated with an 80% of survival (4 of 5 children) [187].

Biodistribution experiments performed by Shi et al. in mice demonstrate that intravenous injection of mitochondria is efficient for delivering mitochondria to diverse tissues, including heart, liver, kidney, muscle and brain [168]. All these organs display increased ATP content once mitochondria are incorporated, being able to enhance the locomotor activity measured through forced swimming test aimed to determine the anti-fatigue properties of therapeutic agents [168]. Even mice carrying a 1-Methyl-4-phenil-1,2,3,6-tetrahydropyridine (MTPT)-induced PD-like phenotype and exposed to mitochondrial transplants show a significant improvement on their behavioral abilities, measured through T-turn in pole, T-LA in pole and Rotarod tests [168].

Zhang et al. have highlighted the pro-regenerative effects of mitochondrial transplantation in stroke. They show that muscle-derived autologous mitochondria injected intraventricularly provoke an overall increase in the number of mitochondria in rats’ brain and reduced oxidative stress in penumbra [190]. Incorporation of mitochondria increases SOD and glutathione peroxidase (GSH-Px) activity accompanied by reduced lipid peroxidation and formation of oxygen and nitrogen-derived reactive species. These changes correlate with poor co-localization of classic apoptotic and neuronal markers and reduces cleaved-caspase-3 expression; the right ischemic hemispheres limit reactive astrogliosis and active the neurogenic process at the boundary of ischemic area [190]. Additionally, transplanted rats present a reduction in the volume of brain infarct regions, while improvements on motor functions were significant as determined through different neurobehavioral scores [190].

In summary, there are several factors that place current therapeutic strategies a step behind mitochondrial transfer, most of them connected with their inability to reverse mitochondrial dysfunction. Thus, pharmacological approaches and naturally isolated antioxidants became mostly an alternative to counteract the consequences but not the origin of this state. Altogether, pre-clinical and clinical evidence demonstrate technical, biological, and medical solvency in the use of isolated cell-free mitochondria as the future therapeutic agents.

## Risk and controversies behind mitochondrial transfer

### Concern regarding mitochondrial-nuclear genome incompatibility

It has been largely explored that nuclear-encoded proteins are transported to the mitochondria including proteins of the electron transport chain or enzymes that manage ROS production such as SOD1 [192]. Thus, mitochondria transfer arises as a potential concern for the probable incompatibility that could emerge from the crosstalk between the nucleus and the mitochondria that might affect critical biological process such as mitochondrial respiration. Even that mitochondrial genome is highly conserved, it is still recommended that mitochondria transplantation consider donors and acceptors and certain grades of compatibilities such as same ethnical descent.

### Ethical and legal issues in mitochondrial transfer

Mitochondrial transfer creates ethical and theoretical issues because it involves the altering of genetic material from different people. Mitochondrial transfer may also produce genetically modified babies with improved qualities, which is another cause for concern. The United Kingdom and, recently, Australia are the only countries with laws allowing mitochondrial donation to prevent the transmission of severe mitochondrial diseases such as Leigh Syndrome [191, 192]. These laws were established based on a UK-developed methodology that has already received enough scientific validation to allow clinical trials [192]. Although clinical advancement in this field is vital, compelling arguments exist that any move toward clinical research must be carefully considered and addressed.

## Mitochondria replacement therapy: Are there projections toward brain degeneration?

There are more than ∼700 disease-associated mitochondrial DNA mutations (mitomap.org). These include severe conditions recognized by an inappropriate bioenergetic profile for organs with elevated demands, including nervous tissues. Emerging therapies like Maternal Spindle Transfer (MST) [193], Pronuclear Transfer (PT) [194], and Polar Body Transfer (PBT) [195] seek to provide an alternative to these defects due to their potential to prevent or cure primary mitochondrial dysfunction. Next, we briefly summarize the principle of these technologies and put into perspective some aspects to consider to carry out their translocation toward brain degeneration.

MST relies on removing the nucleus from a donor egg but preserving its cytoplasmic components, thus subsequently introducing the nucleus from the mother´s egg cell, fertilizing with the father´s sperm, and then transferring to the mother´s uterus for normal gestation [193]. By contrast, PT involves the transfer of pronuclei from one zygote to another, maintaining the prerequisite of fertilizing a healthy donated egg cell (containing donated mitochondria) with the male parent sperm [194]. On the other hand, PBT entails the removal of the first polar body from nonfertilized oocytes to be then transferred into enucleated donor oocytes or the removal of the second polar body once fertilization has taken place to replace it for the female pronucleus in the donor zygote [195]. These techniques preserve the genetic background of the mother but, at the same time, leave aside pathological mitochondria.

Mitochondrial replacement therapy is still maturing, and its translocation toward neurodegenerative disorders is an ongoing challenge. Paine et al. discuss significant technological challenges, including heteroplasmy and reversal, incompatibility and alloreactivity, inapplicability onto human cells, and the accumulation of mitochondria-related mutations in aged persons [196]. Pioneering studies performed by Ma et al. centered on determining the long-term developmental, reproductive, and genetic consequences of mitochondrial replacement in rhesus macaques (*Macaca mulatta*), and their longitudinal investigation suggests that these features remain unaffected. Nevertheless, one of four analyzed monkeys showed high heteroplasmy levels increasing up to 17%, while two others presented up to 33% of paternal mitochondrial DNA contribution [197]. Rationally, there are many factors behind the maturation and progression of these technologies to implement them as a possible treatment for brain degeneration, including a considerable increase in preclinical evidence, the viability of clinical set ups, cost-effectiveness, and social and political aspects. Lastly, these technologies should be projected based on preventing and curing heritable neurodegenerative diseases caused by deleterious mutations in mitochondrial DNA, not those involving mitochondrial dysfunction as a secondary event.

## Conclusion and future perspectives

In acute brain injuries such as stroke, a leading cause of death and adult disability worldwide, robust evidence supports the regenerative potential of mitochondrial transfer. After the injury, neurons release impaired mitochondria to induce mitochondrial transfer from neighboring cells in a Miro1-dependent manner [198]. The release of mitochondria seems to respond to challenges like acidosis, oxidative stress, N-methyl-D-aspartate, and glutamate, increasing the number of damaged mitochondrial units in nervous tissue and cerebrospinal fluid [198]. Analogously, transferring exogenous mitochondria via site-specific injection or systemic administration has proven to promote neuron survival, enhance motor performance and reduce infarct area in brain ischemic rats [199]. Mice models moved similarly, showing that extracellular mitochondria injected in the peri-infarct cortex result in up-regulation of cell-survival-related signals as regulated through CD38 signaling [156]. Besides, patients with subarachnoid hemorrhage showed good neurological recovery when mitochondria with high membrane potential were detected in their cerebrospinal fluid [200].

The differences concerning acute brain injury and degeneration are substantial. Neurodegenerative disorders are long-term diseases with a broad spectrum of molecular and cellular defects, including mitochondrial dysfunction. Once centered on mitochondrial deficiencies, states of deregulation and/or dysfunction denote abnormalities at different levels, involving respiratory complexes, uncontrolled production of oxygen-derived species, a breaking in the appropriate balance between fusion and fission dynamics, deregulation of lipid peroxidation and diverse mitophagy-related pathways leading to an increase in the number of superfluous mitochondrial units, among many others. Consequently, an essential question arises: how feasible it is to treat these abnormalities through a unique therapeutic approach? In this context, mitochondrial transfer stands out as a promising therapeutic strategy for neurodegenerative abnormalities associated with mitochondrial dysfunction.

Since benefits of mitochondrial transplantation are not replicable through the administration of synthetic ADP and ATP or even mitochondria carrying DNA damage, it is tempting to speculate whether exogenous mitochondria can act not only as an acute energy source but also controlling the long-term expression of mitochondrial constituents in recipient cells. Given the invasive nature and potential complications of intraventricularly transplanted mitochondria, methodologies centered in increasing their entry towards the nervous system, are required. For example, approaches aimed to increase the absorption via intranasal delivery, permeabilizing the blood–brain barrier, or reducing their unspecific homing or accumulation in non-targeted regions.

Another technical aspect associated with removing mitochondria from the intracellular environment is given by the overload of calcium, leading to permeability transition pore formation with the subsequent swelling and rupture of mitochondrial membranes [201]. Since mitochondrial swelling has place at micromolar calcium concentrations, it is unlikely that mitochondria exposed to physiological extracellular concentrations remain wholly viable once at circulation or interstitial tissue containing concentrations oscillating between 1.2 and 2 mM [202]. Research aimed at stabilizing and preserving the structure of mitochondria under conditions that replicates those observed in physiological fluids are not fully detailed yet but, and it might optimize the internalization and functionality of mitochondrial transplants in diverse systems, including the nervous system.

Lastly, our laboratory recently discussed the therapeutic potential of stimulating the mitochondrial transfer-related enzymatic machinery [151]. Brain degenerative disorders implies the continuing loss of neurons, each one with a proper pattern of progression. However, there are several high-resistance neuroanatomical regions that remain intact in advanced stages. It is tempting to speculate whether activating endogenous mitochondrial transfer in healthy brain regions might rescue diseased ones [151]. Deepening alternative strategies might reinforce current challenges and issues behind administering exogenous mitochondria.

## Key definitions

1. **Spontaneous mitochondrial transfer.** Naturally occurring intercellular mechanisms for transferring mitochondria from a donor to a recipient cell. The best-known ways include extracellular vesicles, tunneling nanotubes, and Gap junctions.
2. **Artificial mitochondrial transfer.** Also called Mitoception comprises a procedure aimed at isolating mitochondria from a donor cell to incorporate them into recipient cells in vitro utilizing different centrifugation steps.
3. **Mitochondria injection.** It represents an experimental approach that isolates mitochondria for subsequent in vivo administration through different methods, such as intranasal, intraperitoneal, intravenous, and site-specific delivery.
4. **Mitochondrial replacement therapy.** Also called mitochondrial donation, MRT are novel technologies that prevent the maternal transmission of mitochondria DNA diseases. Thus, these are classified as reproductive in vitro fertilization technologies methods.

## Data Availability

Not applicable.

## Abbreviations

OMM: Outer mitochondrial membrane
IMM: Inner mitochondrial membrane
IMS: Intermembrane space
TOM: Translocase of the outer membrane
TIM: Translocase of the inner membrane
MIM: Mitochondrial import complex
MICOS: Mitochondrial contact site and cristae organizing system
EVs: Extracellular vesicles
TNTs: Transitory tunneling nanotubes
MC: Mitoception
Fis1: Mitochondrial fission 1 protein
Drp-1: Dynamin-related protein 1
Mfn1/2: Mitofusin 1 and 2
OPA1: Optic atrophy type 1
mTOR: Mammalian target of rapamycin
AMPK: AMP-activated protein kinase
PTEN: Phosphatase and tensin homolog
PINK1: PTEN-induced kinase
PSEN1/2: Presenilin 1 and 2
MEF2D: Myocyte enhancer factor 2D
PGC1α: Peroxisome proliferator-activated receptor-γ coactivator 1 α
PGC3β: Peroxisome proliferator-activated receptor-γ coactivator 3 β
TFAM: Mitochondrial transcription factor
Nrf1/2: Nuclear respiratory factor 1 and 2
VCP: Valosin-containing protein
SOD1, Cu: Zn superoxide dismutase 1
C9: C9orf72
OPTN: Optineurin
FUS: Fused in sarcoma
H3K4me3: Histone H3 trimethylated on lysine 4
DCX: Doublecortin
PPARγ: Peroxisome proliferator- activated receptor gamma
APP: Amyloid precursor protein
Aβ: Amyloid β
EAE: Experimental autoimmune encephalomyelitis
AD: Alzheimer´s disease
PD: Parkinson´s disease
HD: Huntington´s disease
ALS: Amyotrophic lateral sclerosis
sALS: Sporadic amyotrophic lateral sclerosis
hALS: Hereditary amyotrophic lateral sclerosis
MS: Multiple sclerosis
FA: Friedreich´s ataxia
LB: Lewy bodies
Mt-DNA: Mitochondrial DNA
MTPT: 1-Methyl-4-phenyl-1,2,3,6-tetrahydropyridine
6-OHDA: 6-Hydroxydopamine
8-OHdG: 8-Hydroxy-2´-deoxyguanosine
GSH-Px: Glutathione peroxidase
SNpc: Substantia nigra pars compacta
UPS: Ubiquitin-protease systems 26S and 20S
MPP^+^: 1-Methyl-4-phenylpyridium
iPSC: Inducible pluripotent stem cells
EPC: Endothelial progenitor cells
NSC: Neural stem cells
MSC: Mesenchymal stem/Stromal cells
OLs: Oligodendrocytes
CNS: Central nervous system
Treg: Regulatory T CD4 cells
PBMCs: Peripheral blood nuclear cells
GvHD: Graft versus Host Disease
MST: Maternal spindle transfer
PT: Pronuclear transfer
PBT: Polar body transfer
